# Postoperative short‐term outcomes of minimally invasive versus open esophagectomy for patients with esophageal cancer: An updated systematic review and meta‐analysis

**DOI:** 10.1111/1759-7714.13413

**Published:** 2020-04-20

**Authors:** Naeem M. Akhtar, Donglai Chen, Yuhuan Zhao, David Dane, Yuhang Xue, Wenjia Wang, Jiaheng Zhang, Yonghua Sang, Chang Chen, Yongbing Chen

**Affiliations:** ^1^ Department of Thoracic Surgery, School of Medicine The Second Affiliated Hospital of Soochow University Suzhou China; ^2^ Department of Thoracic Surgery, Shanghai Pulmonary Hospital, School of Medicine Tongji University Shanghai China

**Keywords:** Esophageal cancer, esophagectomy, minimally‐invasive surgery

## Abstract

**Background:**

We performed a systematic review and meta‐analysis to synthesize the available evidence regarding short‐term outcomes between minimally invasive esophagectomy (MIE) and open esophagectomy (OE).

**Methods:**

Studies were identified by searching databases including PubMed, EMBASE, Web of Science and Cochrane Library up to March 2019 without language restrictions. Results of these searches were filtered according to a set of eligibility criteria and analyzed in line with PRISMA guidelines.

**Results:**

There were 33 studies included with a total of 13 269 patients in our review, out of which 4948 cases were of MIE and 8321 cases were of OE. The pooled results suggested that MIE had a better outcome regarding all‐cause respiratory complications (RCs) (OR = 0.56, 95% CI = 0.41–0.78, *P* = <0.001), in‐hospital duration (SMD = −0.51; 95% CI = −0.78−0.24; *P* = <0.001), and blood loss (SMD = −1.44; 95% CI = −1.95−0.93; *P* = <0.001). OE was associated with shorter duration of operation time, while no statistically significant differences were observed regarding other outcomes. Additionally, subgroup analyses were performed for a number of different postoperative events.

**Conclusions:**

Our study indicated that MIE had more favorable outcomes than OE from the perspective of short‐term outcomes. Further large‐scale, multicenter randomized control trials are needed to explore the long‐term survival outcomes after MIE versus OE.

## Introduction

Esophageal cancer is the seventh most common cause of cancer‐related death globally.^1^ The overall five‐year survival is below 20%.^2^, ^3^ The main course of treatment is surgical resection, which is usually combined with chemotherapy or chemo‐radiotherapy for locally advanced tumors.^4^ Conventional surgical treatment involves open esophagectomy (OE) using transthoracic or transhiatal approaches which are associated with high morbidity and mortality. Respiratory complications (RCs) are common with OE and can increase the risk of death up to 20%.^5^, ^6^, ^7^ In recent decades, minimally invasive esophagectomy (MIE) has become an alternative to OE. MIE encompasses a number of techniques including total MIE (tMIE), hybrid minimally invasive esophagectomy (hMIE) and robotic surgery.^8^

Given the technical complexity of MIE, a number of concerns exist regarding the benefits of MIE compared with OE in terms of postoperative complications and short‐term mortality. On one hand, even though a number of previously performed studies have established MIE as a relatively safe procedure in terms of post‐operative outcomes,^9^, ^10^, ^11^, ^12^, ^13^ on the other, studies performed by Seesing *et al*. and Mariette *et al*. state the opposite.^14^, ^15^

With a number of emerging studies regarding MIE and OE in recent years, there has been a lack of a systematic study to investigate the short‐term outcomes after MIE versus OE. Furthermore, a detailed and updated meta‐analysis concerning the two approaches might help surgeons with their surgical decisions. Ergo, the purpose of this systematic review and meta‐analysis was not only to use the latest and largest population‐based data to extensively compare and summarize the postoperative complications after MIE versus OE for esophageal cancer, but also to clarify whether MIE could improve the post‐operative outcomes and overall survival of patients with esophageal cancer.

## Methods

### Literature search strategy

This study was performed according to Preferred Reporting Items for Systematic Reviews and Meta‐Analysis (PRISMA) guidelines. Literature was identified by searching databases including PubMed, EMBASE, Web of Science and Cochrane Library up to June 2019 without language restrictions. The search terms used for literature identifications include “esophageal carcinoma, esophageal cancer, esophagectomy, minimally invasive esophagectomy, open esophagectomy and thoracoscopic laproscopic esophagectomy”.

### Eligibility criteria for literature selection

Literature included in the study had to meet the following criteria: (i) studies comparing MIE with OE; (ii) studies published in English only; (iii) studies including at least 20 or more patients; (iv) studies with assigned NOS (Newcastle‐Ottawa quality assessment scale) score of seven or higher; (iv) prospective, randomized controlled trials or retrospective studies only; and (v) studies where full text was available.

### Data extraction and quality assessment

Literature included in the study was independently assessed for methodological quality purposes (N.A and D.D). First, the titles and abstracts were screened to assess the eligibility of included literature, and then the full text was reviewed. Any discrepancies were resolved in discussion with a third author (C.D). The information recorded for each study is given in Table 1.

**Table 1:** Detailed characteristics of included studies

								ASA classification				TNM staging							
No.	Authors (year)	Country or Region	Study design	Intervention	No. of cases	Sex ratio (M/F)	Median age, years (IQ range) mean ± SD	1	2	3	4	0	1	2	3	4	Pathology (adeno/squam/others)	Neoadjuvant therapy (chemo‐radio /chemo)	NOS score
1	Mariette *et al*. (2019)^15^	France	RCT	hMIE OE	103 104	88/15 87/17	59 (23–75) 62 (41–78)	25 34	61 58	17 12	0 0	NA NA	18 19	30 33	50 48	NA§ NA§	57/46/0 66/38/0	36/41 30/45	9
2	Straatman *et al*. (2017)^16^	The Netherlands	RCT	MIE OE	59 56	43/16 46/10	61.8 ± 8.4 62.3 ± 8.4	10 15	34 32	14 08	01 01	0 1	4 4	26 22	5 4	NA NA	35/24/0 36/19/1	52/4 54/5	9
3	Kinjo *et al*. (2011)^17^	Japan	Retrospective	TLE TE OE	72 34 79	58/14 29/5 70/9	62.7 ± 7.4 64.2 ± 8.8 63.3 ± 8.6	35 15 36	3 19 41	0 0 02	NA NA NA	NA NA NA	21 11 18	26 7 27	16 9 20	9 7 14	0/71/1 3/31/0 3/71/5	NA NA NA	8
4	Sarkaria *et al*. (2018)^18^	USA	Prospective	rMIE OE	64 106	53/11 91/15	61 (45–82) 63 (28–83)	NA NA	09 15	51 84	04 07	13 20	22 25	15 33	14 27	NA NA	59/4/0 98/7/1	47/1 85/2	9
5	Safranek *et al*. (2010)^19^	UK	Prospective	tMIE hMIE OE	41 34 46	25/16 28/6 38/8	64 (41–74) 63 (44–76) 60 (44–77)	NA NA NA	NA NA NA	NA NA NA	NA NA NA	2 2 0	7 2 6	17 14 11	15 16 29	NA NA NA	23/17/1 29/3/2 43/3/0	0/34 0/27 0/34	8
6	Paireder *et al*. (2018)^20^	Austria	RCT	MIE OE	14 12	10/4 10/2	64.5 (40–75) 62.5 (49–77)	NA NA	NA NA	NA NA	NA NA	1 2	4 4	2 2	6 3	1§ 1§	10/4/0 11/1/0	0/9 0/7	8
7	Sihag *et al*. (2016)^21^	USA	Retrospective	MIE OE	814 2966	658/156 2492/474	63.3 ± 10.7 63.2 ± 10.2	NA NA	NA NA	NA NA	NA NA	NA NA	NA NA	NA NA	NA NA	NA NA	NA NA	NA NA	7
8	Schoppmann *et al*. (2010)^22^	Austria	Prospective	MIE OE	31 31	25/6 21/10	61.5 (35.7–74.8) 58.6 (33.7–76.8)	14 15	13 11	04 05	NA NA	NA NA	10 4	4 9	14 15	1 2	17/14/0 12/19/0	NA NA	8
9	Klevebro *et al*. (2018)^23^	Sweden	Prospective	MIE OE	201 165	162/39 132/33	67 (33–83) 65 (36–82)	NA NA	NA NA	NA NA	NA NA	NA NA	19 28	13 34	119 93	32 5	153/41/7 120/42/3	125/20 55/59	8
10	Perry *et al*. (2009)^24^	USA	Retrospective	LE OE	21 21	18/3 17/4	69 ± 8 61 ± 9	(1–2 = 13)† (1–2 = 13)		(3–4 = 8)† (3–4 = 8)		NA NA	NA NA	NA NA	NA NA	NA NA	NA NA	NA NA	7
11	Seesing *et al*. (2017)^14^	The Netherlands	Retrospective	MIE OE	433 433	335/58 335/58	64 ± 9.0 64 ± 8.7	80 65	271 287	82 81	NA NA	NA NA	26 24	86 82	310 311	11§ 17§	305/128/0 311/122/0	375/21 376/21	9
12	Mass *et al*. (2013)^25^	The Netherlands	RCT	MIE OE	14 13	10/4 12/1	65 (56–75) 62 (52–74)	NA NA	NA NA	NA NA	NA NA	NA NA	NA NA	NA NA	NA NA	NA NA	13/1/0 11/2/0	NA NA	8
13	Glatz *et al*. (2017)^26^	Germany	Retrospective	hMIE OE	60 60	49/11 52/8	61 (42–92) 61 (44–84)	(1–2 = 36)† (1–2 = 33)		(3–4 = 24)† (3–4 = 27)		(0–1 = 35)‡ (0–1 = 27)‡		9 14	14 14	1 5	46/14/0 47/13/0	12/35 12/38	9
14	Tang *et al*. (2018)^27^	China	Retrospective	MIE (nCRT) MIE (nCT) OE (nCT)	76 42 57	64/12 33/9 51/6	61 (44–790 61 (46–730 60 (41–73)	24 13 19	48 27 36	04 02 02	0 0 0	NA NA NA	NA NA NA	NA NA NA	47 28 37	29§ 14§ 20§	NA NA NA	NA NA NA	7
15	Lee at al. (2011)^28^	Taiwan	Prospective	tMIE hMIE OE	30 44 64	30/0 43/1 61/3	59.7 ± 10.32 59.7 ± 11.17 56.5 ± 11.60	NA NA NA	NA NA NA	NA NA NA	NA NA NA	2 12 7	3 13 17	11 14 25	12 5 14	2 1 1	1/29/0 1/43/0 5/59/0	NA NA NA	7
16	Bonavina *et al*. (2016)^29^	Italy	Retrospective	TE OE	80 80	46/34 71/9	61.5 (53–70) 63.5 (55–68)	15 21	56 47	09 12	0 0	NA NA	25 15	25 22	23 31	7 12	9/68/3 63/15/2	31¶ 17¶	8
17	Hamouda *et al*. (2009)^30^	UK	Prospective	LE OE	26 24	25/1 23/1	62 60	NA NA	NA NA	NA NA	NA NA	1 0	0 1	4 1	19 18	2§ 3§	21/4/1 21/3/0	NA NA	7
18	Kauppi *et al*. (2014)^31^	Finland	Prospective	MIE OE	74 79	59/15 68/11	66 (51–85) 63 (39–82)	NA NA	NA NA	NA NA	NA NA	NA NA	NA NA	28 25	44 54	0 0	NA NA	3/55 12/59	7
19	Guo *et al*. (2013)^32^	China	RCT	TE OE	111 110	68/43 72/38	57.3 ± 11.8 60.8 ± 12.4	NA NA	NA NA	NA NA	NA NA	NA NA	24 31	80 74	7 5	NA§ NA§	NA NA	NA NA	7
20	Sihvo *et al*.^33^	Finland	Retrospective	MIE OE	150 150	119/31 119/31	63.9 (9.2) 64.3 (8.9)	NA NA	NA NA	NA NA	NA NA	NA NA	NA NA	NA NA	NA NA	NA NA	122/27/10 263/138/30	26/73 31/61	8
21	Pham *et al*. (2010)^34^	USA	Retrospective	TLE OE	44 46	41/3 33/13	63 ± 8.6 61 ± 10.7	(1–2 = 12)† (1–2 = 17)		(3–4 = 32)† (3–4 = 29)		0 0	6 7	14 13	18 18	2 1	34/8/0 34/6/2	NA NA	8
22	Scarpa *et al*. (2015)^35^	Italy	Retrospective	hMIE OE	34 34	27/7 6/25	62 (52–70) 64 (56–70)	5 4	22 17	07 13	NA NA	(0–1‐2 = 29) (0–1‐2 = 29)		(3–4 = 5)‡ (3–4 = 5)‡			24/10/0 24/10/0	22¶ 22¶	8
23	Biere *et al*. (2012)^36^	The Netherlands	RCT	MIE OE	59 56	43/16 46/10	62 (34–75) 62 (42–75)	10 15	34 32	14 08	01 01	NA NA	NA NA	NA NA	NA NA	NA NA	24/35/0 36/19/1	54/5 52/4	9
24	Parameswaran *et al*. (2013)^37^	UK	Prospective	tMIE LE OE	36 31 19	24/12 13/8 15/4	64 (45–84) 67 (48–79) 64 (51–77)	NA NA NA	NA NA NA	NA NA NA	NA NA NA	6 1 0	6 5 0	13 12 8	10 13 11	0 0 0	22/8/5 27/3/0 16/3/0	23¶ 27¶ 17¶	8
25	Noble *et al*. (2012)^38^	UK	Prospective	MIE OE	53 53	43/10 45/8	66 (45–85) 64 (36–81)	4 10	44 32	05 11	NA NA	2 0	0 1	9 15	42 33	0§ 4§	47/4/1 48/3/0	1/12 2/9	9
26	Burdall *et al*. (2014)^39^	UK	Retrospective	LE MIE OE	184 67 83	151/33 48/19 67/16	64.8 (39–79) 65.4 (36–79) 63.9 (43–77)	NA NA NA	NA NA NA	NA NA NA	NA NA NA	6 3 1	32 37 12	25 9 10	119 18 60	2§ 0§ 0§	167/14/3 53/7/0 74/8/1	0/158 0/23 0/76	8
27	Dolan *et al*. (2013)^40^	USA	Retrospective	MIE OE	82 64	65/17 55/9	67 (60–76) 69 (63–75)	1 0	28 16	47 35	03 04	NA NA	NA NA	31 23	48 33	NA NA	NA NA	74¶ 39¶	8
28	Hsu *et al*. (2013)^41^	Taiwan	Retrospective	TE OE	66 63	61/5 58/5	58.8 ± 10.4 60 ± 11.3	NA NA	NA NA	NA NA	NA NA	NA NA	24 15	14 12	25 33	3§ 3§	NA NA	0/10 0/14	7
29	Kanekiyo *et al*. (2017)^42^	Japan	Retrospective	TE OE	65 65	56/9 58/7	66 (62–70) 66 (61–70)	16 14	45 47	04 04	0 0	(0–1 = 24) (0–1 = 24)			(2–3‐4 = 41)‡ (2–3‐4 = 41)‡		NA NA	0/37 0/35	8
30	Rinieri *et al*. (2016)^43^	France	Prospective	MIE OE	70 70	59/11 54/16	61.1 ± 9 61 ± 9	9 14	48 40	13 16	0 0	15 15	22 23	15 11	17 20	1 1	50/20/0 55/15/0	NA NA	8
31	Thomson *et al*. (2010)^44^	Australia	Prospective	TE OE	165 56	134/31 45/11	68 (36–84) 65 (42–82)	(1–2 = 120)† (1–2 = 30)		(3–4 = 45)† (3–4 = 26)		NA NA	51 5	46 16	68 35	NA NA	128/37/0 48/8/0	NA NA	8
32	Yerokun *et al*. (2016)^45^	USA	Retrospective	MIE rMIE OE	1077 231 2958	905/172 195/36 2474/484	57 (64–70) 57 (64–70) 57 (64–70)	NA NA NA	NA NA NA	NA NA NA	NA NA NA	194 52 494	350 72 812	149 31 443	291 63 852	6§ 1§ 12§	861/216/0 186/45/0 2305/653/0	643/0 157/0 800/0	8
33	Zingg *et al*. (2009)^46^	Australia	Prospective	MIE OE	56 98	45/11 71/27	66.3 (1.3) 67.8 (1.1)	NA NA	NA NA	NA NA	NA NA	15 14	9 15	21 33	11 27	NA NA	46/10/0 65/29/4	40¶ 48¶	8

adeno; adenocarcinoma; ASA, American Society of Anesthesiologists; chemo, chemotherapy; chem‐radio, chemo‐radiotherapy; hMIE, Hybrid minimally invasive esophagectomy; IQ, interquartile; LE, laparoscopic‐assisted esophagectomy; M/F, Male/Female; NA, not available; nCRT, neoadjuvant chemo‐radiotherapy; nCT, neoadjuvant chemotherapy; NOS, Newcastle‐Ottawa quality assessment scale; OE, open esophagectomy; RCT, randomized controlled trial; rMIE, robotic‐assisted minimally invasive esophagectomy; SD, standard deviation; squam; squamous cell carcinoma; TE, thoracoscopic esophagectomy; TLE, thoracoscopic laparoscopic esophagectomy; tMIE, total minimally invasive esophagectomy.

† The ASA classification data for multiple stages is provided together.

‡ The TNM staging data for multiple stages is provided together.

§ Mention of size or direct extension of primary tumor only.

¶ Not specified whether the neoadjuvant therapy was chemo‐radiotherapy or chemotherapy.

### Definition of study endpoints

In total, we discussed 11 endpoints in our study: one primary and 10 secondary endpoints. All‐cause respiratory complications (RCs) were chosen to be discussed as the primary endpoint. These RCs included atelectasis, pneumonia, acute respiratory distress syndrome (ARDS), pleural effusion, pneumothorax and respiratory insufficiency. The details of 10 secondary endpoints are given below. All‐cause cardiac complications (CCs) which included cardiac arrest, myocardial infarction, atrial & ventricular dysrhythmia, congestive heart failure and pericarditis; all‐cause anastomotic leakage (AL) defined as full thickness GI defect involving esophagus, anastomosis, staple line, or conduit irrespective of presentation or method of identification; total length of in‐hospital stay; total operation time; total blood loss; R0 resection; 30‐day mortality; 90‐day mortality; all‐cause in‐hospital mortality; and reoperation rate.

### Statistical analysis

SPSS software was used for general data analysis. Data was extracted and entered into review manager. Continuous variables were expressed as median and interquartile ratio or range, and the mean and SD were estimated from the available data. The Mantel‐Haenszel method for dichotomous data was used. Fixed or random‐effects models were used in this study. Forest plots were provided to illustrate pooled odds ratios (ORs), and corresponding 95% confidence intervals (CIs). Cochran's *Q* test and Higgins *I* ^*2*^ were used to test the heterogeneity of different studies. A *P*‐value of less than 0.1 was considered significant. Heterogeneity was interpreted according to the thresholds outlined in the Cochrane Handbook. With significant heterogeneity, a pooled effect was calculated with a random‐effects model; otherwise, a fixed‐effects model was applied. The reasons for interstudy heterogeneity were explored by using subgroup analysis. We also conducted sensitivity analysis by omission of each single study to evaluate stability of the results. Publication bias was assessed by using funnel plots.

## Results

### Selection of eligible studies

**Figure 1:**
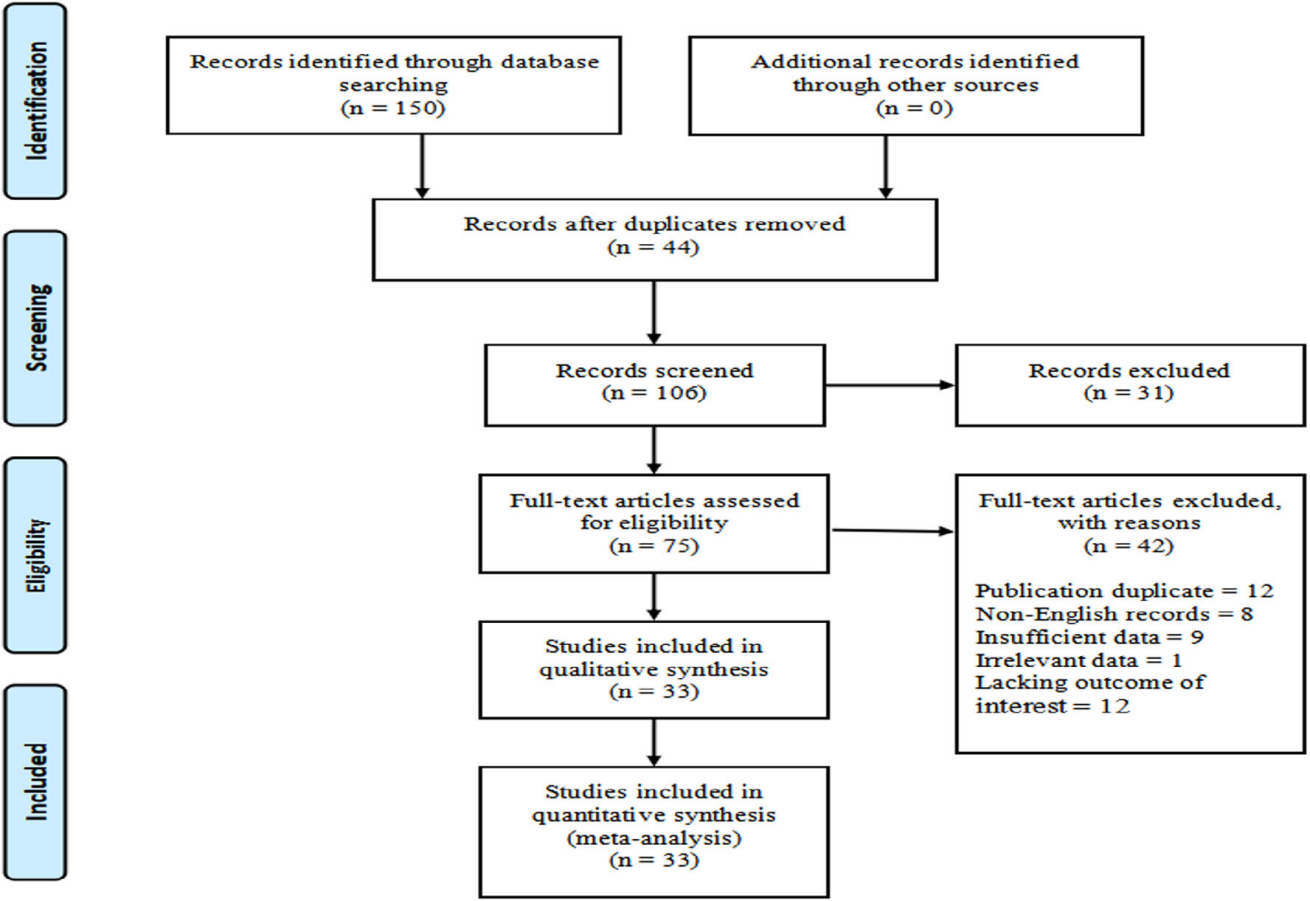
PRISMA flowchart of literature search strategy.

The PRISMA flowchart diagram is shown in Figure 1. In summary, our literature search strategy initially identified 150 articles. Finally, 33 articles qualified to be included in our meta‐analysis study.

### Characteristics of included literature

A total of 13 269 patients were included in this meta‐analysis study, out of which 4948 cases were of MIE and 8321 cases were of OE. Table 1 provides detailed characteristics of the articles included. In summary, six studies had a RCT study design, 12 had a prospective study design and the remaining 15 had a retrospective study design.

### Primary outcome: All‐cause RCs

**Figure 2:**
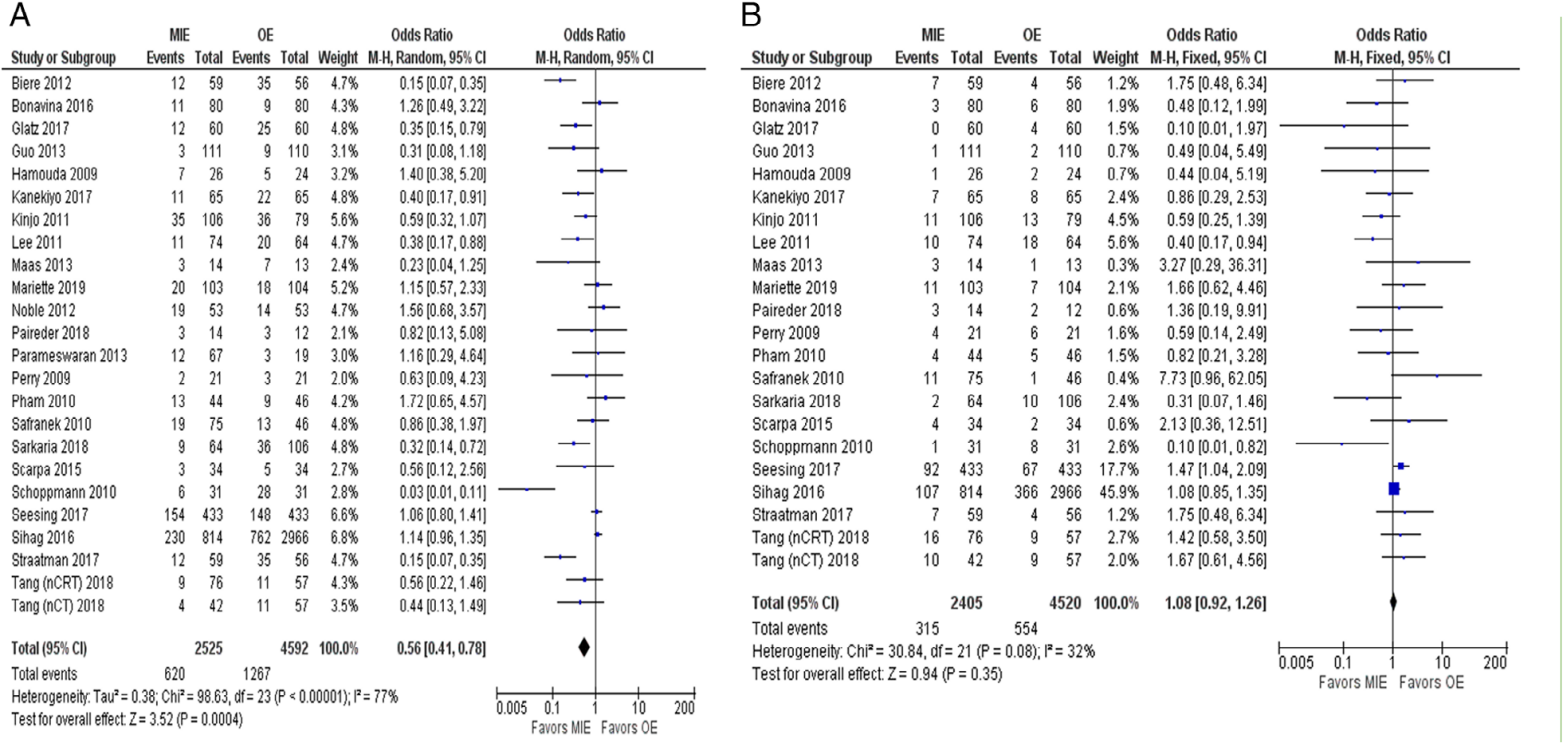
(**a**) Forest plot of all‐cause RCs. (**b**) Forest plot of all‐cause AL.

A total of 24 studies^14^, ^15^, ^16^, ^17^, ^18^, ^19^, ^20^, ^21^, ^22^, ^24^, ^25^, ^26^, ^27^, ^28^, ^29^, ^30^, ^32^, ^34^, ^35^, ^36^, ^37^, ^38^, ^42^ with 7117 patients were involved in the analysis of all‐cause RCs. Figure 2a shows that the patients who underwent MIE experienced less postoperative RCs as compared to those who underwent OE (OR = 0.56; 95% CI = 0.41, 0.78; *P* = <0.001). Test of heterogeneity showed considerable heterogeneity (*I* ^*2*^ = 77% and *P* = <0.001)*. S*ubgroup analyses were conducted to explore potential sources of that heterogeneity (Table 2). The pooled ORs of most subgroups were not markedly changed by the study characteristics. However, the subgroup analysis by intervention type showed considerable significance for tMIE/OE (*P* = <0.001; *I* ^*2*^ = 91%) as compare to hMIE/OE (*P* = 0.07; *I* ^*2*^ *=* 35%) which was less significant. We also noted the changes in statistical heterogeneity in the subgroup analysis of different institutes and facilities (single center, *I* ^*2*^ *=* 64%; multicenter, *I* ^*2*^ *=* 83%), initial inclusion period (<2008, *I* ^*2*^ *=* 68%; ≥2008 *I* ^*2*^ *=* 88%), study design (RCT, *I* ^*2*^ = 74%; prospective, *I* ^*2*^ = 79%; retrospective, *I* ^*2*^ = 54%), and NOS score (7, *I* ^*2*^ = 56%; 8, *I* ^*2*^ = 74%; 9, *I* ^*2*^ = 87%). Sensitivity analysis was conducted by omission of each single study to evaluate the stability of results indicating an unaffected pooled OR. The funnel plots displaying the publication bias of all cause RCs is shown in Figure S2b.

**Table 2:** Subgroup analyses of all‐cause RCs of MIE and OE

		Test of association			Test of heterogeneity	
Variable	Studies	OR	95% CI	*P*‐value	I^2^ (%)	*P*‐value
Total	24	0.56	0.41–0.78	<0.001	77	<0.001
Publication year						
<2016	13	0.51	0.29–0.90	<0.001	72	0.02
≥2016	11	0.61	0.42–0.90	<0.001	77	0.01
No. of cases						
<100	9	0.52	0.22–1.24	0.001	69	0.014
>100	15	0.57	0.40–0.81	<0.001	80	0.002
Research region						
The Netherlands	4	0.29	0.08–1.07	<0.001	92	0.06
The UK	4	1.19	0.72–1.96	0.79	0.00	0.49
The USA	4	0.84	0.40–1.74	0.02	71	0.63
China (Mainland)	3	0.45	0.24–0.88	0.78	0.00	0.02
Italy	2	1.1	0.45–2.24	0.38	0.00	0.99
Japan	2	0.51	0.32–0.84	0.45	0.00	0.007
Austria	2	0.14	0.00–4.13	0.004	88	0.25
Miscellaneous regions (Germany, France, Taiwan)	3	0.55	0.25–1.21	0.05	67	0.14
Institutes/facilities						
Single center	14	0.56	0.36–0.88	0.01	64	<0.001
Multicenter	10	0.57	0.36–0.90	0.02	83	<0.001
Initial inclusion period						
<2008	12	0.64	0.38–1.08	<0.001	68	0.09
≥2008	12	0.50	0.32–0.77	<0.001	82	0.002
Study design						
RCT	6	0.33	0.14–0.79	0.001	74	0.01
Prospective	7	0.52	0.23–1.20	<0.001	79	0.13
Retrospective	11	0.79	0.59–1.05	0.02	54	0.11
Intervention						
tMIE/OE	7	0.33	0.16–0.68	<0.001	91	0.002
hMIE/OE	17	0.68	0.51–0.90	0.07	35	0.008
Neoadjuvant therapy						
With	13	0.59	0.37–0.92	<0.001	76	0.02
Without	11	0.52	0.30–0.90	<0.001	77	0.02
NOS score						
7	7	0.66	0.39–1.11	0.03	56	0.12
8	10	0.64	0.39–1.06	<0.001	74	0.08
9	7	0.48	0.24–0.97	<0.001	87	0.04

CI, confidence interval; hMIE, hybrid minimally invasive esophagectomy; NOS, Newcastle‐Ottawa quality assessment scale; OR; odds ratio; RCT, randomized controlled trial; tMIE, total minimally invasive esophagectomy.

### Secondary endpoints

**Figure 3:**
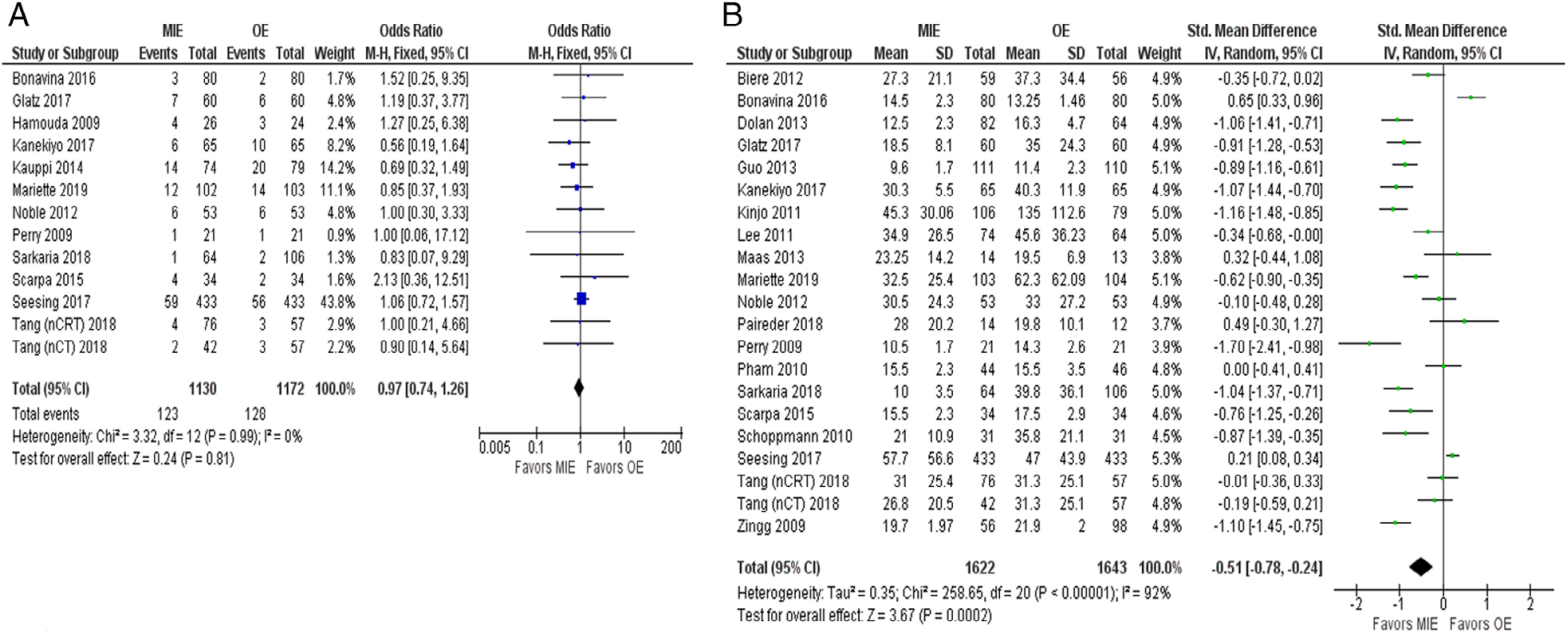
(**a**) Forest plot of all‐cause CCs. (**b**) Forest plot of in‐hospital stay.

A total of 22 studies^14^, ^15^, ^16^, ^17^, ^18^, ^19^, ^20^, ^21^, ^22^, ^24^, ^25^, ^26^, ^27^, ^28^, ^29^, ^30^, ^32^, ^34^, ^35^, ^36^, ^38^, ^42^ with 6925 patients were included in the analysis of all‐cause AL, which showed low level of heterogeneity (*P* = 0.08, *I* ^*2*^ = 32%) and no statistical difference between MIE versus OE (OR = 1.08; 95% CI = 0.92, 1.26; *P* = 0.35) (Figs 2b, S2c). Data for all‐cause CCs was reported in 13 studies^13^, ^14^, ^18^, ^24^, ^26^, ^27^, ^29^, ^30^, ^31^, ^35^, ^38^, ^42^ with 2302 patients and showed neither heterogeneity (*P* = 0.99, *I* ^*2*^ = 0%), nor statistically significant difference between MIE or OE (OR = 0.97; 95% CI = 0.74, 1.26; *P* = 0.81) (Figs 3a, S2d).

**Figure 4:**
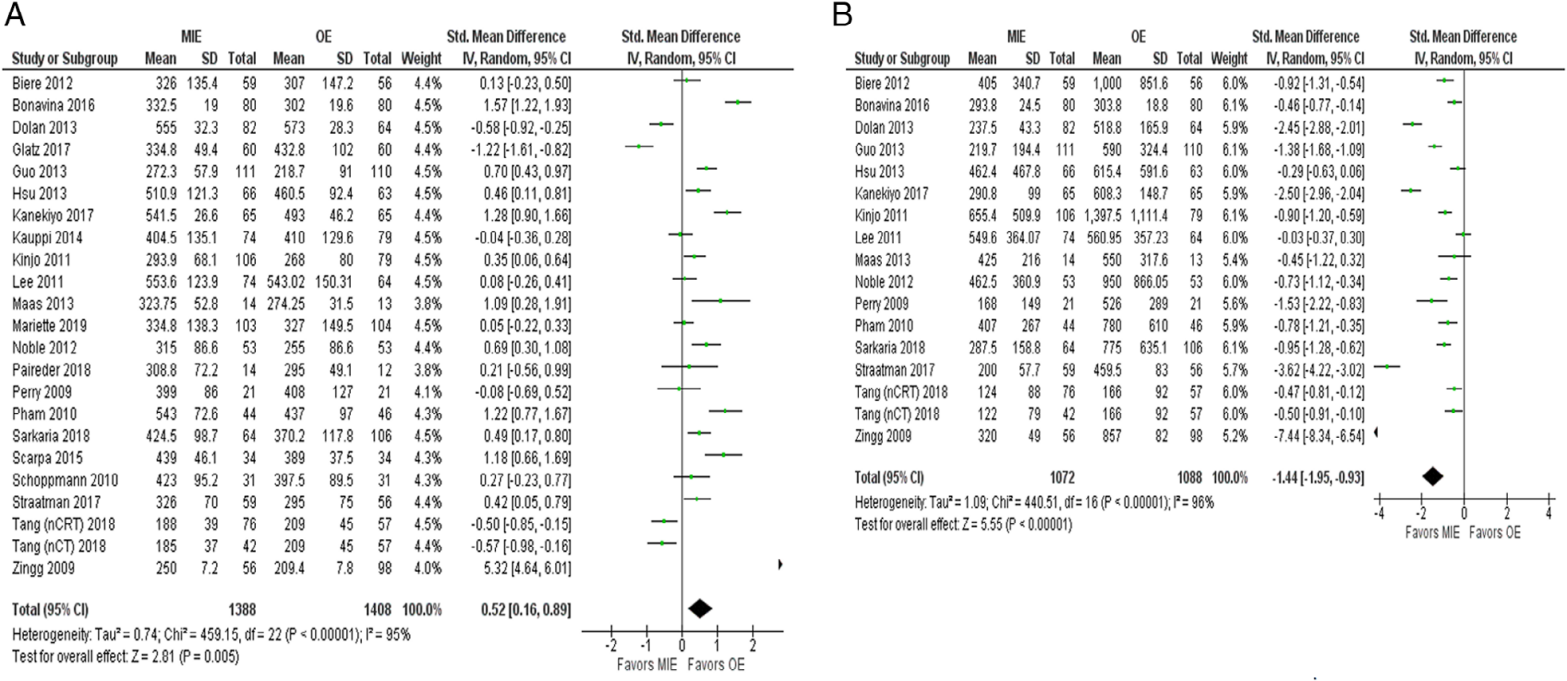
(**a**) Forest plot of total operation time. (**b**) Forest plot of blood loss.

Evaluation of data for total length of in‐hospital stay from 21 studies^14^, ^15^, ^17^, ^18^, ^20^, ^22^, ^24^, ^25^, ^26^, ^27^, ^28^, ^29^, ^32^, ^34^, ^35^, ^36^, ^38^, ^40^, ^42^, ^46^ with 3265 patients showed that patients who underwent MIE got to experience less in‐hospital duration compared with those who underwent OE (SMD = −0.51; 95% CI = −0.78, −0.24; *P* = <0.001) (Fig. 3b). Substantial heterogeneity (*P* = <0.001, *I* ^*2*^ = 92%) was found and subgroup analyses were performed to explore the potential source of heterogeneity as shown in Table S1. A total of 23 studies^15^, ^16^, ^17^, ^18^, ^20^, ^22^, ^24^, ^25^, ^26^, ^27^, ^28^, ^29^, ^31^, ^32^, ^34^, ^35^, ^36^, ^38^, ^40^, ^41^, ^42^, ^46^ with 2796 patients included in analyzing the data for total operation time showed that the patients who underwent MIE experienced longer operation time compared to those who underwent OE (SMD = 0.52; 95% CI = 0.16, 0.89; *P* = 0.005) (Fig. 4a and Table S2).

Data for total blood loss gathered from 17 studies^16^, ^17^, ^18^, ^24^, ^25^, ^27^, ^28^, ^29^, ^32^, ^34^, ^36^, ^38^, ^40^, ^41^, ^42^, ^46^ with 2160 patients revealed that MIE resulted in less blood loss in comparison with OE (SMD = −1.44; 95% CI = −1.95, −0.93; *P* = <0.001) (Fig. 4b). The outcome also indicated the presence of substantial heterogeneity (*P* = <0.001, *I* ^*2*^ = 96%) which led us to perform subgroup analyses to analyze the source of heterogeneity (Table S3). Other outcomes such as R0 resection (OR = 1.47; 95% CI =1.13, 1.92; *P* = 0.004), 30‐day mortality (OR = 0.92; 95% CI = 0.69, 1.22; *P* = 0.56), 90‐day mortality (OR = 0.52; 95% CI = 0.29, 0.91; *P* = 0.02), in‐hospital mortality (OR = 0.73; 95% CI = 0.38, 1.41; *P* = 0.35), and the rate of reoperation (OR = 1.30; 95% CI = 0.85, 1.98; *P* = 0.22) showed no significant statistical differences between MIE and OE as shown in Figs S1a‐S1d, S2a.

## Discussion

This study compared the outcomes of OE with both tMIE and hMIE. Due to the complexity of esophagectomy, different types of surgical approaches might lead to different kinds of surgical complications, but the main morbidities remain the same which include RCs, CCs, AL and the aforementioned.

Most of the meta‐analysis studies comparing the outcomes of MIE and OE previously performed were either based on retrospective studies only, or had a small sample size.^11^, ^47^, ^48^, ^49^ Although, Lv *et al*. had a relatively larger sample size of 6025 patients from 20 studies, their study only included literature up to 2016.^12^ Since then, a considerable number of updated studies have been published, showing new findings and discrepancies in their results.^9^, ^13^, ^14^, ^15^, ^16^, ^18^, ^20^, ^23^, ^26^, ^27^, ^33^, ^42^ In contrast, we included 33 studies in total involving 13 269 patients in our meta‐analysis to provide the latest and more robust outcomes comparing MIE and OE.

Postoperative RCs are of great importance and could impact the prognosis of patients, which are also the most frequent morbidity events after esophagectomy. Some previous studies have shown contradictory results regarding the advantages of MIE over OE with respect to postoperative RCs. Two retrospective studies showed no significant differences regarding RCs between two groups.^13^, ^50^ On the other hand, two RCTs showed a significantly lower incidence of respiratory complications after MIE than OE.^36^, ^51^ Pooled data from our study also showed that patients who underwent MIE experienced fewer postoperative RCs compared to those who underwent OE (Fig. 2a). The association of MIE with fewer postoperative RCs could be explained by the elegance of the MIE operation procedure which decreases surgical trauma to the chest wall and does less harm to pulmonary tissues.

The results from our study showed that MIE was associated with a longer operative time as compared to OE. These results were consistent with other recently published studies and could be attributed to the technical difficulty in MIE and a limited operating space for surgeons to perform the delicate procedure.^16^, ^18^, ^42^ Data analyses also demonstrated that patients who underwent MIE experienced shorter postoperative in‐hospital stay and had less in‐operative blood loss, as compared to those who underwent OE. Both these results were in accordance with previous studies and can be associated with the less intrusive nature of MIE.^23^, ^26^, ^36^

Notably, pooled results and subgroup analyses from our study showed no significant correlation between neoadjuvant therapy and improvement of postoperative outcomes, either after MIE or OE.

## Principle findings and limitations

Our meta‐analysis provides strong evidence for the association of MIE with overall better short‐term outcomes (Table 3). When stratified by publication year, initial inclusion period, number of cases, types of surgical intervention, and NOS quality score, the results remained mostly constant. Meanwhile, the heterogeneity in subgroup analyses was shown to be not considerable in general. In addition, with the application of some advanced statistical methods, the results have demonstrated that the outcomes tend to be much more stable with the increasing number of studies over time.

**Table 3:** Summary of the final results of all primary and secondary endpoints

Endpoints	Studies	Cases	OR/SMD	95%CI	*P*‐value	I^2^	*P*‐value	Favors
All‐cause RCs	24	7117	0.56	0.41, 0.78	<0.001	77%	<0.001	MIE
All‐cause AL	22	6925	1.08	0.92, 1.26	0.35	32%	0.08	None
All‐cause CCs	13	2302	0.97	0.74, 1.26	0.81	0%	0.99	None
In‐hospital stay	21	3265	−0.51	−0.78, −0.24	<0.001	96%	<0.001	MIE
Total operation time	23	2796	0.52	0.16, 0.89	0.005	95%	<0.001	OE
Blood loss	17	2160	−1.44	−1.95, −0.93	<0.001	96%	<0.001	MIE
R0 resection	13	2938	1.47	1.13, 1.92	0.004	0%	0.56	None
30‐day mortality	12	7976	0.92	0.69, 1.22	0.56	0%	0.95	None
90‐day mortality	6	1095	0.52	0.29, 0.91	0.02	0%	0.91	None
In‐hospital mortality	8	846	0.73	0.38, 1.41	0.35	0%	0.71	None
Reoperation	10	4767	1.30	0.85, 1.98	0.22	33%	0.14	None

AL, anastomotic leakage; CCs, cardiac complications; CI, confidence interval; MIE; minimally invasive esophagectomy; OE, open esophagectomy; OR; odds ratio; RCs, respiratory complications; SMD, standardized mean difference.

There are several limitations to our study that should also be acknowledged. First, as shown in Table 1, the pathological TNM staging and ASA classification is missing from several included studies, which resulted in undeniable differences in their quality and strength. Second, patients of different ethnical groups were placed together into MIE or OE groups, which would also have effects on the results of this study. Third, different MIE methods (tMIE or hMIE) were used in different included studies, which makes it difficult to more specifically point out if there was any particular MIE technique that was the most beneficial for better outcomes. Fourth, there is also a possibility that patients with beneficial prognostic variables, for example, younger age and less comorbidity, were more readily selected for MIE rather than OE. Finally, even though our study included several RCTs, the lack of larger number of multi‐institutional RCTs might reduce the effectiveness of the research. Consequently, the work definitely needs to be improved when there are more RCTs. Although advanced statistical methods were applied, publication bias was inevitable as shown in Fig. S2.

In conclusion, while OE was associated with shorter operation time and a slightly better surgical clearance of the tumor (R0 resection rates) compared with MIE, MIE was associated with fewer RCs, lesser blood loss, shorter postoperative in‐hospital stay and better overall postoperative outcomes. Further large‐scale, multicenter RCTs are needed to continue to explore further long‐term survival outcomes of patients with MIE and OE.

## Disclosure

The authors report that there are no conflicts of interest.

## Supporting information


**Figure S1.** (**a**) Forest plot of R0 resection; (**b**) forest plot of 30‐day mortality; (**c**) forest plot of 90‐day mortality; and (**d**) forest plot of in‐hospital mortality.

**Figure S2.** (**a**) Forest plot of reoperation; (**b**) funnel plot of all‐cause RCs; (c) funnel plot of all‐cause AL; and (**d**) funnel plot of all‐cause CCs.

**Table S1.** Subgroup analysis of in‐hospital stay between MIE and OE.
**Table S2.** Subgroup analysis of total operation time between MIE and OE.
**Table S3.** Subgroup analysis of blood loss between MIE and OE.
