# Supplementary figures and images for: Postoperative short‐term outcomes of minimally invasive versus open esophagectomy for patients with esophageal cancer: An updated systematic review and meta‐analysis
Source: Thorac Cancer. 2020 Apr 20;11(6):1465–75. doi: 10.1111/1759-7714.13413 (PMC7262946; doi:10.1111/1759-7714.13413)

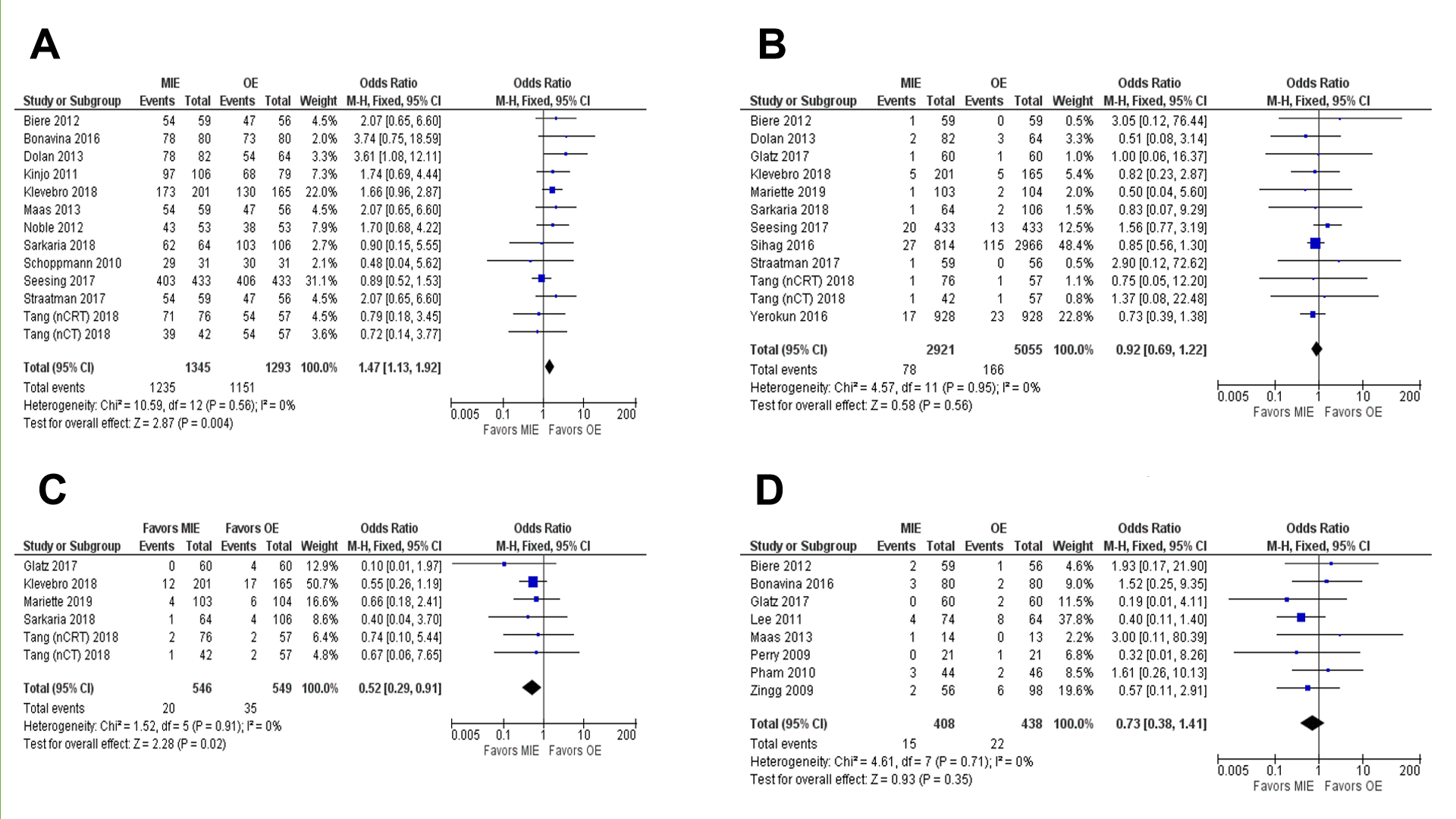

Supplement: Supplementary file 1 — Figure S1. (a) Forest plot of R0 resection; (b) forest plot of 30‐day mortality; (c) forest plot of 90‐day mortality; and (d) forest plot of in‐hospital mortality. [file TCA-11-1465-s001.tif]

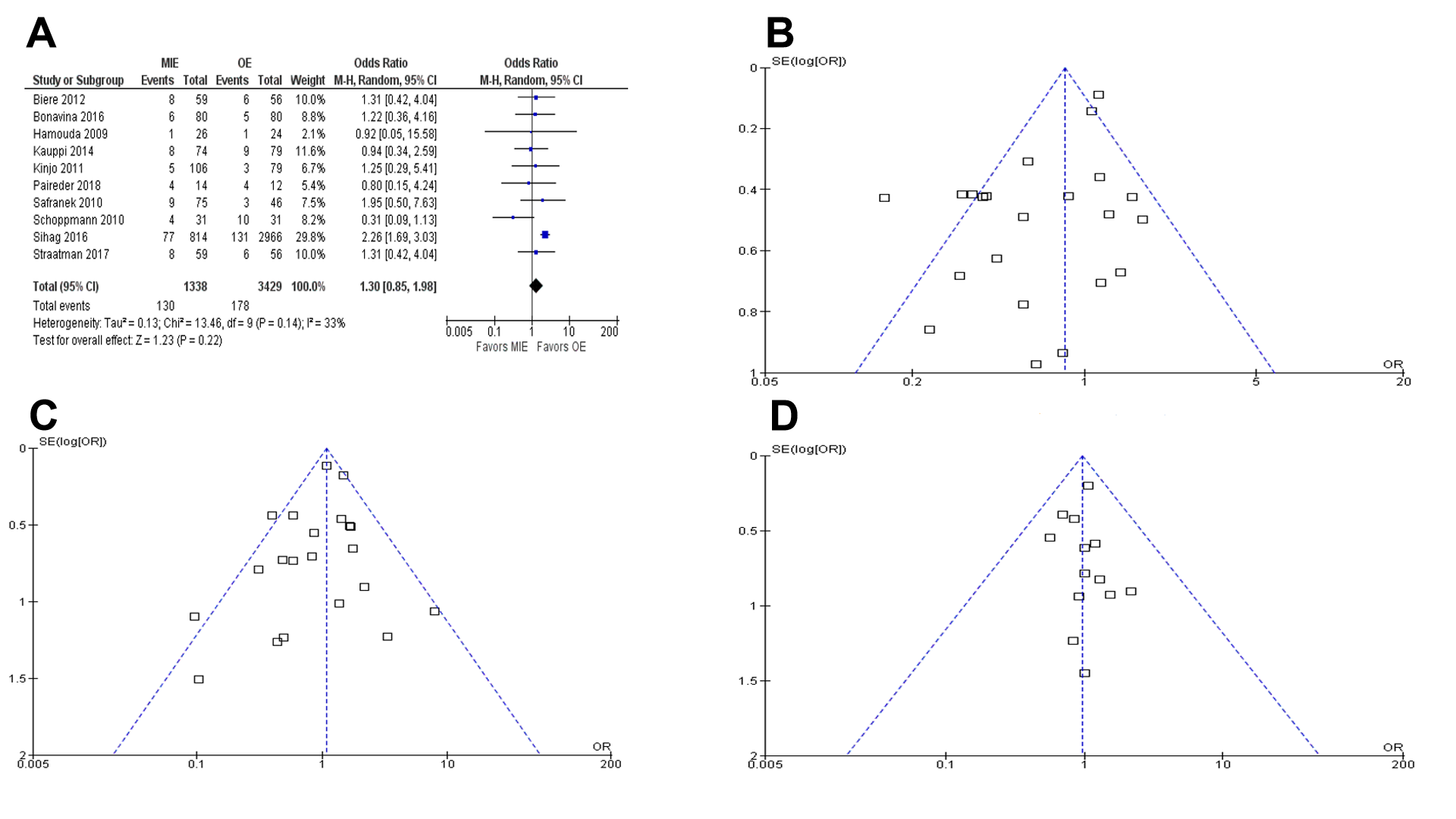

Supplement: Supplementary file 2 — Figure S2. (a) Forest plot of reoperation; (b) funnel plot of all‐cause RCs; (c) funnel plot of all‐cause AL; and (d) funnel plot of all‐cause CCs. [file TCA-11-1465-s002.tif]
