# Supplementary material for: Postoperative short‐term outcomes of minimally invasive versus open esophagectomy for patients with esophageal cancer: An updated systematic review and meta‐analysis
Source: Thorac Cancer. 2020 Apr 20;11(6):1465–75. doi: 10.1111/1759-7714.13413 (PMC7262946; doi:10.1111/1759-7714.13413)
Supplement: Supplementary file 3 — Table S1. Subgroup analysis of in‐hospital stay between MIE and OE. Table S2. Subgroup analysis of total operation time between MIE and OE. Table S3. Subgroup analysis of blood loss between MIE and OE. [file TCA-11-1465-s003.docx]

**Supplemental Table 1 - Sub-group analysis of in-hospital stay between MIE and OE**

| **Variables** | **Studies** | **Test of association** | **Test of heterogeneity** |
| --- | --- | --- | --- |
|  |  | **OR/SMD 95% CI *p*-value** | ***I^2^* (%) *p*-value** |
| **Total** | 21 | -0.51 -0.78, -0.24 <0.001 | 96% <0.001 |
| **Publication year**  <2016  ≥2016 | 12  9 | -0.67 -0.95, -0.40 <0.001  -0.29 -0.71, 0.13 0.17 | 82% <0.001  94% <0.001 |
| **No. of cases**  <100  >100 | 7  14 | -0.40 -0.87, 0.07 0.10  -0.55 -.89, -0.22 0.001 | 80% <0.001  94% <0.001 |
| **Institutes / Facilities**  Single center  Multicenter | 13  8 | -0.41 -0.74, -0.08 0.01  -0.53 -1.07, -0.01 0.05 | 90 <0.001  96 <0.001 |
| **Neoadjuvant therapy**  Yes  No | 12  9 | -0.48 -0.87, -0.10 0.01  -0.53 -0.89, -0.17 0.004 | 94% <0.001  86% <0.001 |
| **Study design**  RCT  Prospective  Retrospective | 5  5  11 | -0.34 -0.75, 0.06 0.09  -0.69 -1.09, -0.28 <0.001  -0.52 -0.94, -0.10 0.01 | 79% <0.001  83% <0.001  94% <0.001 |
| Abbreviations: **RCT**, randomized controlled trial; **OR**; odds ratio; **SMD**, standardized mean difference **CI**, confidence interval. | | | |

**Supplemental table 2 - Sub-group analysis of total operation time between MIE and OE**

| **Variables**  **Total** | **Studies**  **23** | **Test of association** | **Test of heterogeneity** |
| --- | --- | --- | --- |
|  |  | **OR/SMD 95% CI *p*-value** | ***I^2^* (%) *p*-value** |
| Total | 23 | 0.52 0.16, 0.89 0.005 | 95% <0.001 |
| **Publication year**  <2016  ≥2016 | 14  9 | 0.74 0.25, 1.22 0.003  0.20 -0.38, 0.77 0.50 | 95% <0.001  95% <0.001 |
| **No. of cases**  <100  >100 | 7  16 | 0.47 -0.13, 1.06 0.12  0.55 0.10, 0.99 0.02 | 88% <0.001  96% <0.001 |
| **Institutes / Facilities**  Single center  Multicenter | 15  8 | 0.38 -0.01, 0.76 0.05  0.82 -0.01, 1.66 0.05 | 93 <0.001  97 <0.001 |
| **Neoadjuvant therapy**  Yes  No | 14  8 | 0.66 0.14, 1.18 0.01  0.26 -0.17, 0.69 0.23 | 96% <0.001  90% <0.001 |
| **Study design**  RCT  Prospective  Retrospective | 6  6  11 | 0.39 0.10, 0.68 0.009  1.10 0.07, 2.12 0.04  0.28 -0.27, 0.83 0.32 | 68% 0.009  98% <0.001  96% <0.001 |
| Abbreviations: **RCT**, randomized controlled trial; **OR**; odds ratio; **SMD**, standardized mean difference **CI**, confidence interval. | | | |

**Supplemental table 3 - Sub-group analysis of blood loss between MIE and OE**

| **Variables** | **Studies** | **Test of association** | **Test of heterogeneity** |
| --- | --- | --- | --- |
|  |  | **OR/SMD 95% CI *p*-value** | ***I^2^* (%) *p*-value** |
| Total | 17 | -1.44 -1.95, -0.93 <0.001 | 96% <0.001 |
| **Publication year**  <2016  ≥2016 | 11  6 | -1.48 -2.15, -0.80 <0.001  -1.39 -2.23, -0.55 0.001 | 97% <0.001  96% <0.001 |
| **No. of cases**  <100  >100 | 4  13 | -0.78 -1.19, -0.37 <0.001  -1.64 -2.26, -1.02 <0.001 | 56% 0.08  97% <0.001 |
| **Institutes / Facilities**  Single center  Multicenter | 10  7 | -0.79 -1.18, -0.41 <0.001  -2.45 -3.70, -1.19 <0.001 | 91 <0.001  98 <0.001 |
| **Neoadjuvant therapy**  Yes  No | 9  8 | -2.09 -3.02, -1.17 <0.001  0.74 -1.10, -0.38 <0.001 | 98% <0.001  85% <0.001 |
| **Study design**  RCT  Prospective  Retrospective | 4  4  9 | -1.60 -2.66, -0.53 0.003  -2.22 -4.01, -0.43 0.02  -1.08 -1.60, -0.56 <0.001 | 95% <0.001  99% <0.001  94% <0.001 |
| Abbreviations: **RCT**, randomized controlled trial; **OR**; odds ratio; **SMD**, standardized mean difference **CI**, confidence interval. | | | |
